# Gut microbiome and metabolism alterations in schizophrenia with metabolic syndrome severity

**DOI:** 10.1186/s12888-024-05969-9

**Published:** 2024-07-24

**Authors:** Hongxia Zhao, Guang Zhu, Tong Zhu, Binbin Ding, Ahong Xu, Songyan Gao, Yufan Chao, Na Li, Yongchun Chen, Zuowei Wang, Yong Jie, Xin Dong

**Affiliations:** 1 School of Medicine, Shanghai University, Shanghai, 200444 China; 2 Zhanjiang Institute of Clinical Medicine, Central People’s Hospital of Zhanjiang, Zhanjiang, 524045 China; 3 Hongkou Mental Health Center, Shanghai, 200083 China; 4 School of Life Sciences, Shanghai University, Shanghai, 200444 China; 5 Institute of Translational Medicine, Shanghai University, Shanghai, 200444 China; 6 Department of Pharmacy, The First Naval Hospital of Southern Theater Command, Zhanjiang, 524000 China; 7 Clinical Research Center for Mental Health, School of Medicine, Shanghai University, Shanghai, 200083 China

**Keywords:** Multi–omics, Schizophrenia, Metabolic syndrome, Biomarker

## Abstract

**Background:**

Schizophrenia (SCZ) patients undergoing antipsychotic treatment demonstrated a high prevalence and harmful effects of metabolic syndrome (MetS), which acted as the major cause of cardiovascular disease. The major clinical challenge is the lack of biomarkers to identify MetS episodes and prevent further damage, while the mechanisms underlying these drug-induced MetS remain unknown.

**Methods:**

This study divided 173 participants with SCZ into 3 groups (None, High risk, and MetS, consisting of 22, 88, and 63 participants, respectively). The potential biomarkers were searched based on 16S rRNA gene sequence together with metabolism analysis. Logistic regression was used to test the effects of the genus-metabolites panel on early MetS diagnoses.

**Results:**

A genus-metabolites panel, consisting of *Senegalimassilia*, sphinganine, dihomo-gamma-linolenoylcholine, isodeoxycholic acid, and MG (0:0/22:5/0:0), which involved in sphigolipid metabolism, fatty acid metabolism, secondary bile acid biosynthesis and glycerolipid metabolism, has a great discrimination efficiency to MetS with an area under the curve (AUC) value of 0.911 compared to the None MetS group (*P* = 1.08E-8). Besides, *Senegalimassilia*, 3-Hydroxytetradecanoyl carnitine, isodeoxycholic acid, and DG(TXB2/0:0/2:0) distinguished between subgroups robustly and exhibited a potential correlation with the severity of MetS in patients with SCZ, and may act as the biomarkers for early MetS diagnosis.

**Conclusions:**

Our multi–omics study showed that one bacterial genus-five lipid metabolites panel is the potential risk factor for MetS in SCZ. Furthermore, *Senegalimassilia*, 3-Hydroxytetradecanoyl carnitine, isodeoxycholic acid, and DG(TXB2/0:0/2:0) could serve as novel diagnostic markers in the early stage. So, it is obvious that the combination of bacterial genus and metabolites yields excellent discriminatory power, and the lipid metabolism provide new understanding to the pathogenesis, prevention, and therapy for MetS in SCZ.

**Supplementary Information:**

The online version contains supplementary material available at 10.1186/s12888-024-05969-9.

## Background

Schizophrenia (SCZ), characterized by psychotic symptoms such as hallucinations, delusions, cognitive symptoms, and social and occupational decline, remains an etiological and therapeutic challenge. Among the treatment, second-generation antipsychotics (SGAs) are recommended for maintenance treatment in schizophrenia priority because of their effectiveness on reducing symptoms and improving social function [1, 2]. However, many of these drugs, especially clozapine and olanzapine, have severely troubling side-effects, with the weight gain and metabolic syndrome (MetS) happed most obviously [3, 4].

MetS is a constellation of a series of metabolic risk factors characterized by a combination of abdominal obesity (also known as central obesity), high blood pressure (BP), low high-density lipoprotein cholesterol (HDL-C), elevated triglycerides (TG), and hyperglycemia [5, 6]. SGAs have been reported to cause weight gain, lipid disturbance, and insulin resistance, highly contributing to MetS development [7]. Clinical treatment gives priority to the combination of different antipsychotic drugs, this brings a high possibility of MetS, especially clozapine and olanzapine [8]. As one of the most severe side effects, MetS cause high exposure to cardiovascular diseases (CVD) [5, 9], and it is the leading cause of death in SCZ [10, 11]. Additionally, MetS significantly affects patients’ cognitive function and eventually causes mental disability in cognition [12, 13]. Owing to the above damages, MetS led to a bad poor medication compliance, which in turn aggravated the difficulty in SCZ clinical treatment. However, the current criteria neither describe physiologic risk factors completely, nor reflect real-time physical conditions. It is reported that there are some potential biomarkers in blood, urine or microorganism, such as acyl-carnitines [14], folic acid [15], *Eubacterium rectale* and *Roseburia intestinalis* and their related metabolites short chain fatty acid (SCFA) [16], and other metabolites including carbohydrates, acids, hormones, other organic compounds, proteins, lipids, vitamins, amino acid and metals [17], have potential correlations with certain metabolic parameters and antipsychotic-induced MetS. Unfortunately, there is little evidence to suggest that these biomarkers have a high ability to distinguish MetS in SCZ, and a combined multi-omics strategy has not been applied in biomarker discovery for SCZ with MetS. Furthermore, biomarkers for the early diagnosis of MetS in SCZ are lacking, hindering more rational medication, and their translation into the clinical setting is still inadequate. Moreover, evidence regarding the mechanisms inherent to antipsychotic drugs that contribute to an increased MetS risk is also lacking. Therefore, urgent further studies are needed to bridge this gap.

MetS is composed of a series of metabolism disorders, including sugars, lipids, and other small molecule metabolites [9]. However, it is more like a unified complex network influenced by the host and the numerous internal microorganisms rather than only the host’s metabolites disorder. Many necessary metabolites and gut bacteria are involved in highly relevant activities that are crucial in the well-functioning of human metabolism and play important roles in developing into MetS. For example, *A. muciniphila* has negatively correlated with obesity, type 2 diabetes and hypertension [18]. Further, microbiome-based interventions are gaining popularity to treat and prevent metabolic disorders [19]. Bacterial metabolite, such as SCFAs, trimethylamine, secondary bile acids, or components of the bacterial cell wall lipopolysaccharide (LPS), are closely related to dyslipidemia, insulin resistance, and MetS, and the resulting atherosclerotic plaques remain the major contribution to CVD and glucose metabolism disorder and obesity [20, 21]. Gut microbial dysbiosis and the metabolites aberrant greatly interplay. However, how did gut microbiome–metabolome axis disturbed and what was the pathological mechanism of abnormal functions when MetS occurs in SCZ remains unknown. Therefore, it is urgent to further identify early diagnostic signatures associated with microbial metabolites to improve therapeutic strategies during MetS development after taking antipsychotics.

Recently, an increased number of studies have shown that multi-omics strategy such as microbiomics and metabolism performed well in the study of disease markers [22, 23]. To decipher the questions above, we performed integrative 16S rRNA gene sequencing and metabolomic analyses in the SCZ cohort to identified MetS-associated microbial species and fecal metabolites that might be related to MetS risk after antipsychotic administration. The discriminative microbial-metabolites panel could distinguish MetS and even the high-risk group from None symptoms of MetS, which could provide a deep insight of disordered functions and mechanism on MetS in patients with SCZ as well as alert and improve therapeutic strategies.

## Methods

### Subject recruitment

All participants signed a written informed consent before any procedure was performed. This study recruited 276 patients of SCZ regardless of gender from Hong Kou District Mental Health Center, Shanghai, and collected fecal samples. Two senior psychiatrists diagnosed SCZ by a structured psychiatric interview using the tenth edition of the International Classification of Diseases (ICD-10) criteria. Specifically, the structured psychiatric interview was a conversation between the doctor and the patient, mainly including psychiatric examination (such as comprehensive disorders of sensation and perception), thinking disorders (such as thinking associations, thinking logical and thinking content), emotional disorders (related to emotional proterties, emotional fluctuation, and emotional coordination), willpower and behavior disorders, intelligence and self-awareness disorders. As previously mentioned, MetS diagnosis was under the definitions of the Chinese Diabetes Society according to Clinical guidelines in China (2022 edition), MetS was determined by the presence of 3 or more of the following metabolic risk factors: ① Abdominal obesity (i.e. central obesity) (Waist circumference ≥ 90 cm in men and ≥ 85 cm in women). ②Fasting plasma glucose ≥ 6.1 mmol/ or 2-h plasma glucose ≥ 7.8 mmol/L, or a history of diabetes mellitus with antidiabetic medication. ③elevated BP (≥ 130/85 mm Hg, 1 mm Hg = 0.133 kPa) or a history of hypertension with antihypertensive medication. ④Increased TG (TG ≥ 1.7 mmol/L, ⑤reduced HDL-C (HDL-C < 1.04 mmol/L). Figure S1 describes the inclusion and exclusion criteria presented in the flow chart of the study design. Fresh stool samples were collected from each participant and immediately frozen at − 80 °C until analysis and avoid freeze–thaw repeat.

### DNA extraction, PCR amplification and sequencing

The E.Z.N.A.® DNA Kit (Omega Bio-tek, Norcross, GA, U.S.) was used to extract microbial community genomic DNA from fecal samples following the manufacturer’s instructions (Shanghai Majorbio Bio-pharm Technology Co., Ltd, Shanghai, China). Briefly, the DNA extract was checked on 1% agarose gel, and DNA concentration and purity were determined with NanoDrop 2000 UV–vis spectrophotometer (Thermo Scientific, Wilmington, USA). The hypervariable region V3–V4 of the bacterial 16S rRNA gene was amplified with primer pairs 338F (5'-ACTCCTACGGGAGGCAGCAG-3') and 806R(5'-GGACTACHVGGGTWTCTAAT-3'). The PCR amplification of the 16S rRNA gene was performed to obtain amplicons and further purified using the AxyPrep DNA Gel Extraction Kit (Axygen Biosciences, Union City, CA, USA).

### 16S rRNA gene sequence analysis

The raw 16S rRNA gene sequencing reads were demultiplexed, and quality was filtered by fastp version 0.20.0 [24] and merged by FLASH version 1.2.7 [25] with the criteria attached in Supplementary Information A.

UPARSE version 7.1 (http://drive5.com/uparse/) was used to cluster operational taxonomic units (OTUs) with 97% similarity cutoff [26], and chimeric sequences were identified and removed. The taxonomy of each OTU representative sequence was analyzed by RDP Classifier version 2.2 [27] against the 16S rRNA database (e.g., Silva v138) using a confidence threshold of 0.7. Different families between groups were analyzed based Mann–Whitney U test (*p*-value < 0.05) to identify discriminative microbial markers across the MetS and NoMetS groups.

PICRUSt was used to standardize the OTU abundance using the greengene id corresponding to each OTU, and the OTU is annotated with COG and Kyoto Encyclopedia of Genes and Genomes (KEGG, http://www.genome.jp/kegg/) functions from the eggNOG database (evolutionary genealogy of genes: Nonsupervised Orthologous Groups, http://eggnog.embl.de/) to obtain the pathway and functional abundance annotations.

### Metabolomics profile analysis

Details of the metabolomics methods were applied as our previously published protocols [28]. Briefly, Freeze-dried stool samples were prepared by homogenization, extraction, and centrifugation after being weighed accurately. Supernatant were analyzed using Agilent 1290 Infinity LC system coupled with Agilent 6545 accurate mass quadrupole time-of-flight (Q-TOF) mass spectrometer (Agilent, USA). Various metabolites that are responsible for the discrimination between groups were filtered by SIMCA-P software (version 14.1, Umetrics AB, Umeå, Sweden) and SPSS. Human Metabolome Database (http://www.hmdb.ca), Metlin (http://metlin.scripps.edu), and Lipid Maps Structure Database were used to identify metabolites. Then, the relative levels of metabolites between the groups are shown in a heat map analysis based on the MetaboAnalyst 5.0 platform (http://www.metaboanalyst.ca). To investigate the related disturbed pathways and function, we performed the differential metabolites using MetaboAnalyst 5.0 platform and QIAGEN’s Ingenuity Pathway Analysis (IPA®, QIAGEN Redwood City, www.qiagen.com/ingenuity). The profile procedure details are attached in Supplementary Information B.

### Multi-omic correlation analysis

Spearman correlations between microbiota and metabolites and clinical parameters were calculated using R (version 3.3.1, pheatmap package). The metabolites origin analysis and the link between the changed microbiota and metabolites were preformed used Sankey network analysis in MetOrigin [29] (http://metorigin.met-bioinformatics.cn/)to explain the statistical correlation as well as specific metabolic reaction and their biological relationship based on KEGG. Furthermore, Cytoscape (Version: 3.9.1) was applied to establish gut microbiota-metabolites-pathways and function network.

### Statistical analysis

Statistical analyses were conducted using Microsoft Excel 2021, SPSS version 21 (SPSS, Chicago, IL, US), and GraphPad Prism version 7. Continuous, normally distributed variables were analyzed by one-way ANOVA analysis. The Kruskal Wallis test was used for non-normal distributed data for the nonparametric analysis, then categorical variables were compared by employed the χ2 test. Different types of variables were annotated below Table 1. A *P*-value of < 0.05 indicates significant differences. Spearman correlations between differential feces metabolites, feces microbiota, and clinical parameters were calculated using R (version 3.3.1, pheatmap package) and SPSS. Logistic regression analysis and receiver operating characteristic (ROC) curves were operated based on SPSS.

**Table 1:** Characteristics of the study cohort

	**None(*n* = 22)**	**High Risk(*n* = 88)**	**METS(*n* = 63)**	**F or χ2 value**	***P*** **value**
**PANSS_classification**				1.076^**e**^	9.52E-01
Positive^a^	3(13.63)	11(12.50)	8(12.70)		
Negative^a^	18(81.82)	75(85.23)	53(84.13)		
None^a^	1(4.55)	2(2.27)	2(3.17)		
**History of SCZ, years**^**b**^	59(14.25)	61.5(11.00)	62(8.00)	1.053^**e**^	5.91E-01
**Gender**				0.953^**e**^	6.39E-01
Female^a^	12(54.55)	45(51.14)	35(44.44)		
Male^a^	10(45.45)	43(48.86)	28(55.56)		
**Years of SCZ**^**c**^	32.77 ± 13.50	30.25 ± 12.73	30.79 ± 11.78	0.359^**d**^	6.99 E-01
**Psychiatry Medications (PMs)**				1.936^**e**^	9.52E-01
Single antipsychotic drug^a^	11(50.00)	47(53.41)	30(47.62)		
Two antipsychotic drug^a^	11(50.00)	39(44.32)	32(50.79)		
There and above drugs ^a^	0(0)	2(2.27)	1(1.59)		
**BMI,kg/m**^**2c**^	20.66 ± 3.49	22.70 ± 3.77	25.91 ± 3.57	23.384^**d**^	1.07E-09
**Hypertension**^**a**^	0(0.00)	20(22.73)	30(47.62)	21.317^**e**^	2.09E-05
**Diabetes**^**a**^	0(0.00)	12(13.64)	24(38.10)	20.033^**e**^	2.71E-05
**Waist, mean ± SD**^**b**^	80.50(7.50)	90.0(14.75)	100.0 ± 13.00	70.115^**e**^	5.95E-16
**Systolic Pressure, mmHg**^**b**^	112.5(15.50)	121(20)	131.00(20.00)	33.377^**e**^	5.65E-08
**Diastolic Pressure, mmHg**^**c**^	71.36 ± 6.40	78.76 ± 8.99	87.16 ± 10.67	29.744^**d**^	8.39E-12
**Fast Blood Glucose,mmol/L**^**b**^	4.62(1.11)	4.99(0.84)	5.62(1.40)	24.538^**e**^	4.69E-06
**Triglyceride (TG), mmol/L**^**b**^	0.94(0.51)	1.24(0.82)	1.95(0.95)	53.0138^**e**^	3.08E-12
**HDL-C, mmol/L**^**b**^	1.59(0.48)	1.32(0.46)	1.05(0.30)	50.156^**e**^	1.28E-11
**LDL-C**^**b**^	2.35(1.10)	2.80(1.15)	2.82(1.51)	1.509^**e**^	4.70E-01
**TC**^**c**^	4.56 ± 0.80	4.70 ± 1.05	4.64 ± 1.11	0.158^**d**^	8.54E-01

^**a**^ n (%), Categorical variables compared by χ^2^ test

^**b**^ median (IQR), non-normal distributed data variables compared by Kruskal Wallis test

^**c**^ mean ± SD, Continuous, normally distributed variables analyzed by one-way ANOVA test

^**d**^ F value in one-way ANOVA test

^**e**^ χ2 value in Kruskal Wallis test and χ2 test

## Results

### Characteristics of the study

Patients with SCZ were enrolled at Hong Kou Mental Health Center through a strict pathological diagnostic and exclusion process. We divided the participants into three groups following the number of diagnostic indicators (NoDI) they met based on MetS diagnosis guidelines: None (NoDI: 0, *n* = 22), High risk (NoDI: 1–2, *n* = 88), MetS (NoDI: ≥ 3, *n* = 63). The key demographic variables, including age, gender, Positive and Negative Syndrome Scale (PANSS) classification [30] (positive/negative type: ≥ 3 items with a score of ≥ 4 in the positive/negative symptom subscale), psychiatry medication quantity (PMs), and body mass index (BMI) and the risk factors of MetS summarized in Table 1. BP, waist, blood glucose, TG, and HDL-C of these patients were increased with the increased NoDI, and the BMI, which was closely associated with MetS [9], also significantly increased. while other variables, such as PANSS classification, age, gender, and PMs, have no difference between groups, indicating that they were not confounding factors between groups.

### Alterations of the gut microbiota in SCZ patients with MetS

**Fig. 1:**
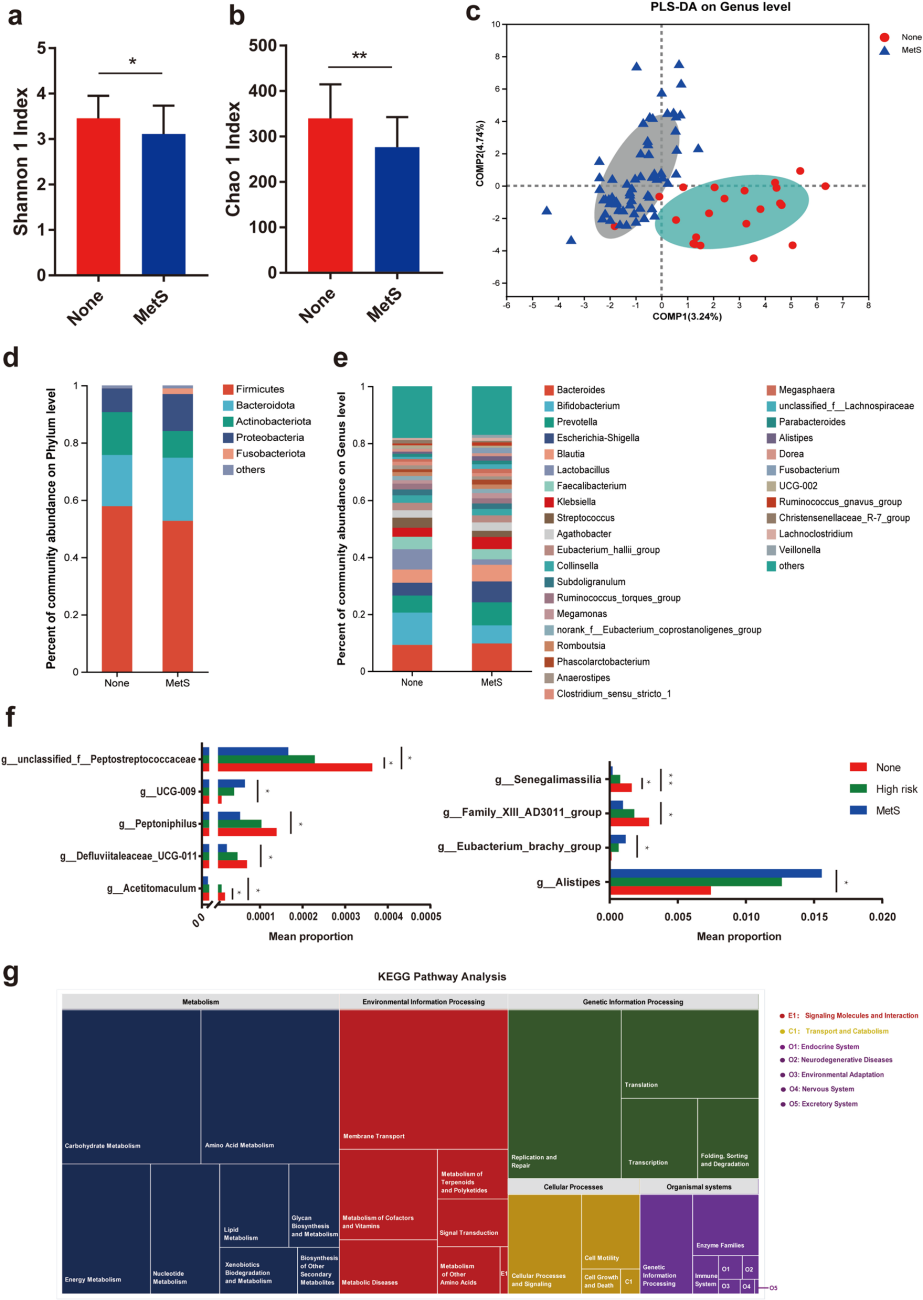
Differential gut microbial characteristics of SCZ with MetS. **a**,** b** The microbial community diversity (Shannon index; **a**, *p* = 0.0367) and richness (Chao 1 index; **b**, *p* = 0.0037) (**c**) Score plot of the PLS-DA constructed based on genus level. **d**,** e** The relative abundance of microbial taxa at phylum and genus levels; phyla or genera with a relative abundance < 1% in each sample are merged into others. **f** Histogram of 9 differential expressed genus gradually changed with MetS under Mann–Whitney U test, *P < 0.05, **P < 0.01. **g** Function prediction analysis using PICRUST based on EggNOG and KEGG

We investigated the alteration in gut microbiota and the functions of gut microbiota that are associated with MetS in SCZ patients. The Chao 1 and Shannon indices, which represent the microbiota richness and diversity in α-diversity analysis based on OUT level, were significantly lower in the MetS group than in the None group (Fig. 1a, b). The score plot of PLS-DA based on genus level, which is a supervised multivariate statistical analysis method for discriminant analysis, showed an obvious separated trend to assess the overall structure of the gut microbiota (Fig. 1c), indicating differences between groups. At the phylum level (Fig. 1d), the MetS group was characterized by a significantly lower abundance of *Firmicutes* and *Actinobacteriota* and higher abundance of *Bacteroidota* and *Proteobacteria* than in the None group. At the genus level (Fig. 1e), dysbiosis in MetS was characterized by an expansion of genera such as *Bacteroides*, *Prevotella* and *Alistipes*, and a decrease in *lactobacillus* and *senegalimassilia*.

Genera were further compared across the three groups to identify those that consistently increased or decreased, in order to ascertain the potential biomarkers that changed with symptom aggravation. This analysis was based on 13 genera that were exclusive to MetS in SCZ compared with the None group previously. Finally, we observed a steady increase in 3 genera and a steady decrease in 6 genera across the three groups. (Fig. 1f**, **Table S1). To further identify the functional features of the gut microbiome with the progress of MetS, function prediction analysis (Fig. 1g) based on PICRUST revealed that variously expressed microbiota mainly involved not only endogenous metabolites, such as carbohydrates, amino acids, lipids, nucleotides, and vitamins but also some xenobiotic biodegradation and metabolism. This indicated that the gradually expressed microbiota between these three groups affect the function of carbohydrate, amino acid, and lipid metabolism and have a high correlation with MetS.

**Fig. 2:**
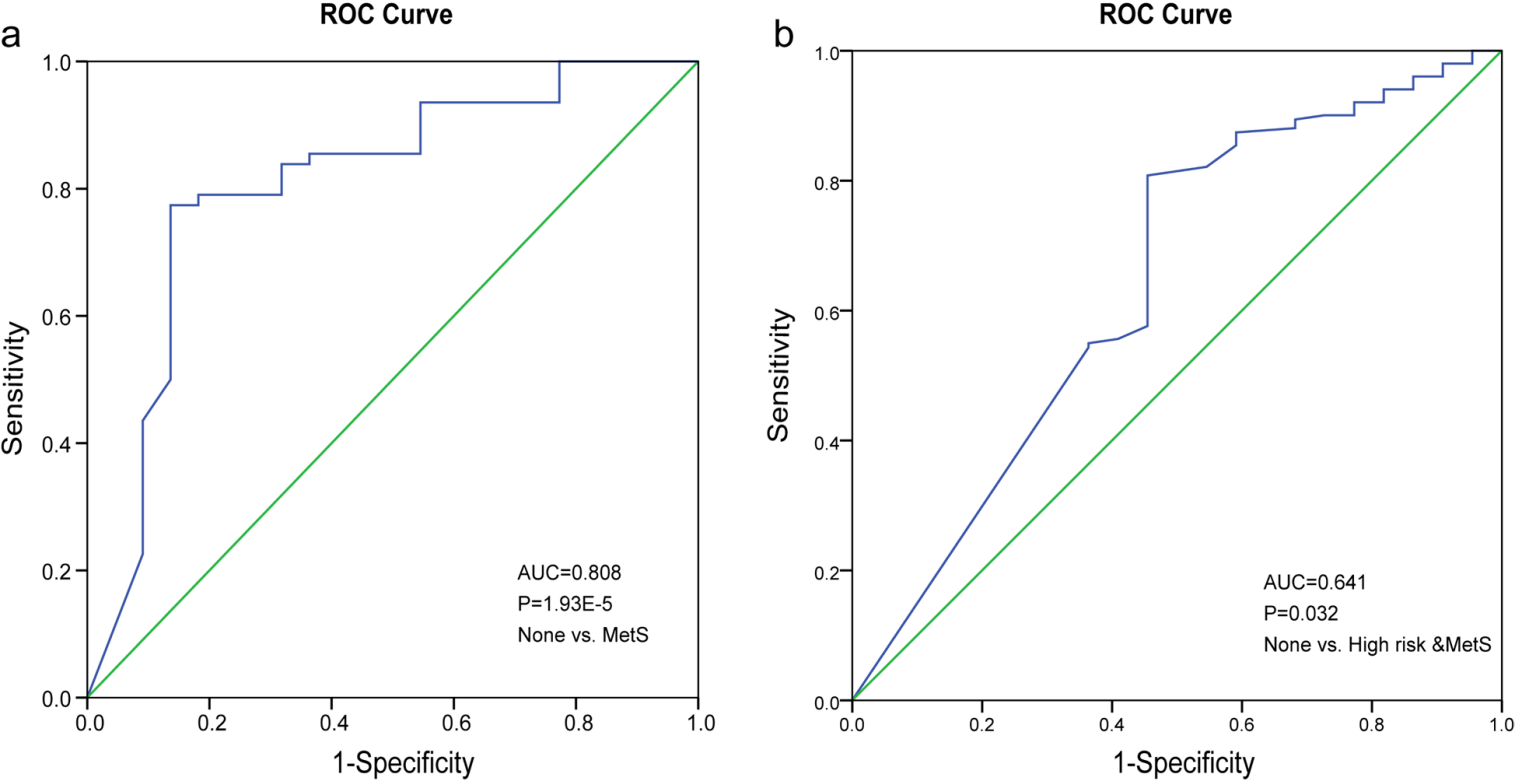
Genera diagnostic outcomes are shown via ROC curves for MetS by logistic regression analysis. **a** Three genera (*Senegalimassilia*, g_unclassified_f_*Peptostreptococcaceae*, *Acetitomaculum*) penal for the classification of patients with SCZ from None group. **b** One genus (*Senegalimassilia*) for distinguish high risk patients of SCZ from None group. None: *n* = 22, High risk: *n* = 88, MetS: *n* = 63

Using logistic regression analysis, we constructed the optimal classifier model of None vs. MetS and None vs. high risk and MetS using these 9 gradually changed genera, and a group of 3 genera (*Senegalimassilia*, *unclassified_f_Peptostreptococcaceae*, *Acetitomaculum*) was selected as the key genera that provided the best discriminatory power by enter-in cross-validation, with AUC values of 0.808 in ROC curves analysis (*p* < 0.001, Fig. 2a). While only genus *Senegalimassilia*, with AUC values of 0.641 in ROC curves analysis (*p* < 0.032, Fig. 2b), found in None vs. high risk and MetS discriminatory analysis, suggesting that genera have potential diagnostic power for SCZ, however, they were not enough for early diagnosing high risk subjects.

### Changes of feces metabolites in SCZ patients with MetS

**Fig. 3:**
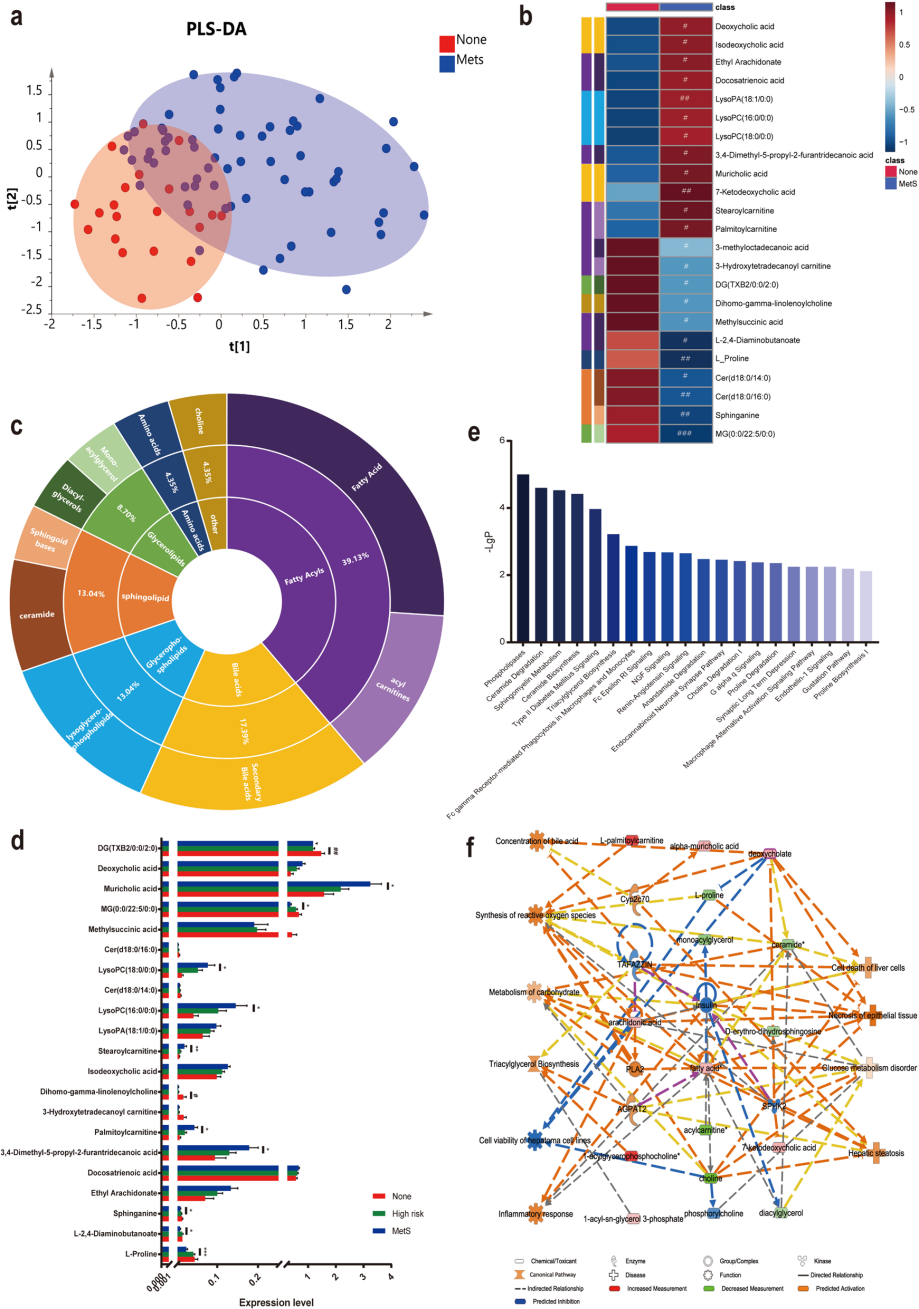
Different expressed metabolites associated with MetS in SCZ. **a** PLS-DA score plot between MetS and None group. **b** Hierarchical clustering correlating levels of fecal metabolites in SCZ with and without MetS visualized as a heatmap. **c** The type classification of 23 changed metabolites. **d** Histogram of 21 changed metabolites with the aggravation of MetS under Mann–Whitney U test,^*^P < 0.05, vs. None, ^**^P < 0.01, vs. None. ^#^*P* < 0.05, vs. High risk, ^##^*P* < 0.01, vs. High risk. **e** The top 20 confidence enriched canonical pathways based on 21 changed metabolites. **f** Function enrichment analysis of 21 changed metabolites under IPA analysis

To further investigate the biological effects of the gut microbiota in different MetS categories, untargeted metabolome profiles was applied on feces samples by liquid chromatography–mass spectrometry. The system stability was conducted by injecting a QC every 18 samples during the whole sample batch. The total ion chromatography of the 10 QC samples demonstrated a good overlap in both positive and negative ion modes (Figure S2 a, b). The RSD values of the peak intensities in the QC samples were used to measure stability. As shown in Fig. S2 c, more than 80% of the RSD values of the QC samples were less than 20%. QC samples in principal component analysis (PCA) (Figure S2 d, e) also displayed a high degree of aggregation. These results demonstrated the robust stability of the proposed method. Then, the entire normalized data were imported into the SIMCA-P program (version 14). There was a clearly separation clusters between None and MetS groups based on the PLS-DA models (Fig. 3a), indicating that MetS has significantly changed at the metabolic levels.

We finally identified 23 metabolites dysregulated in MetS significantly (Table S2, Fig. 3b), with 11 downregulated and 12 upregulated in the MetS group. The same type of metabolites is clustered together generally. Interestingly, amino acids, choline, glycerolipids, and sphingolipid metabolites were downregulated, while bile acids, glycerophospholipids, and acyl carnitines were upregulated in the MetS group (Fig. 3c). Notably, all lysoglycerophospholipids, long-chain fatty acids, and most of the acylcarnitines increased in MetS, while SCFAs belong to the opposite trend. Additionally, 21 metabolites were regarded as symptom-aggravation related and potential markers highly related to MetS development process in SCZ because they changed gradually from the None group to High-risk group. (Fig. 3d). Among them, 10 metabolites changed significantly between the MetS and high-risk groups, while only 2 metabolites changed obviously between high risk and None group, supposed that high-risk group did not reach the diagnosed MetS level, however, the inner molecular changes already appear before the occurrence of clinical symptoms.

We further investigated the related disturbed pathways and function using IPA to obtain better explorations on the internal changes associated with MetS aggravation in SCZ. The top 20 confidence-enriched canonical pathways under the Benjamin-Hochberg test showed in Fig. 3e, and ceramide degradation and biosynthesis, sphingomyelin metabolism, type II diabetes mellitus signaling, triacylglycerol biosynthesis, and endothelin-1 signaling seemed of great importance in the regulation of blood lipids, blood glucose and BP, which are the top three important diagnostic index of MetS. Additionally, the related diseases and functions were displayed in Fig. 3f. Activated and inhibited functions were ultimately affected the activation of liver cell death, epithelial tissue necrosis, glucose metabolism disorder, and hepatic steatosis, and they have a similarity function enrichment with those from changed genera and were consistent with MetS symptoms.

**Fig. 4:**
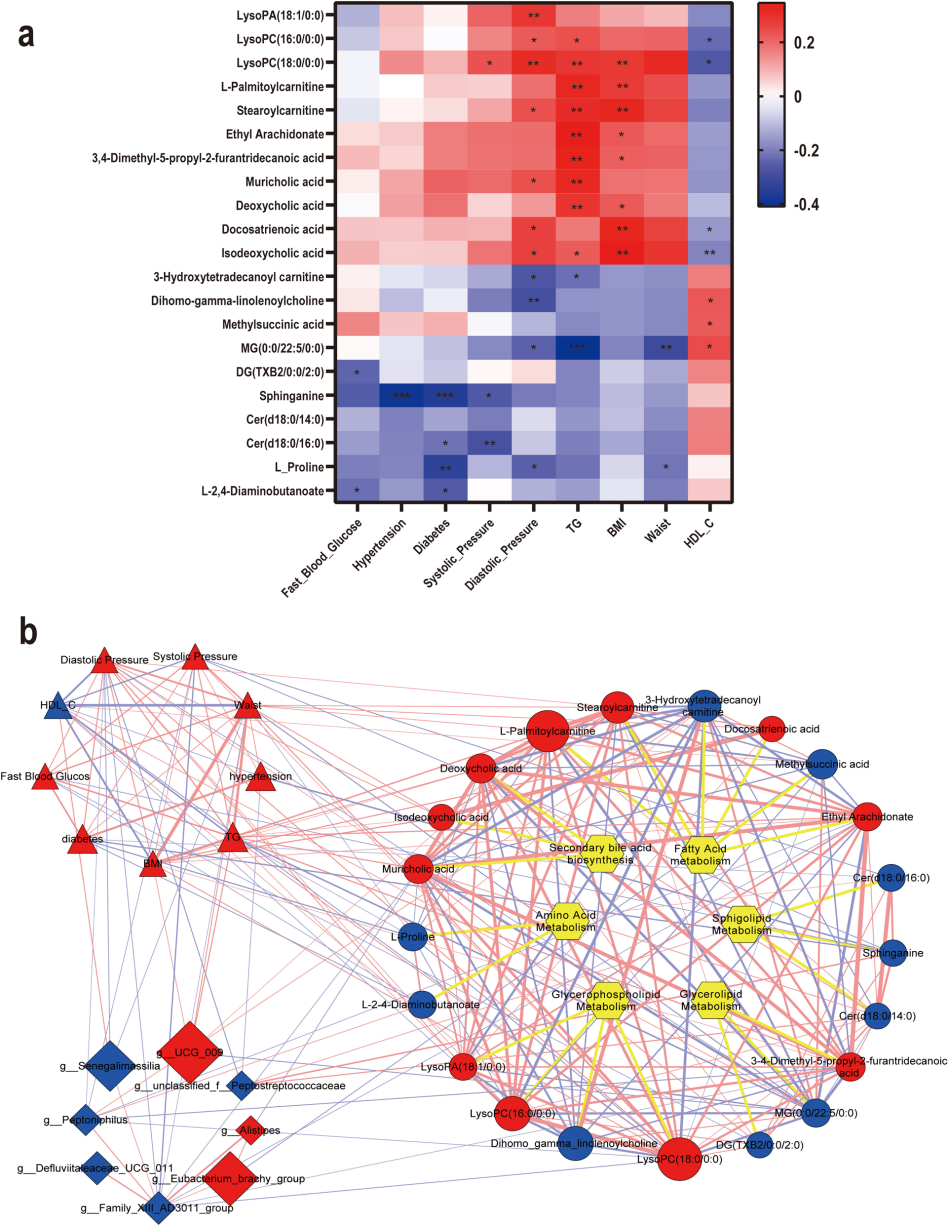
Metabolites interacted with genera and MetS indicators. **a** Spearman correlations between MetS clinical indicators and metabolites. The color represents positive (red) or negative (blue) correlation, and FDRs are denoted as follows: *, *p* < 0.05, **, *p* < 0.01, ***, *p* < 0.001. **b** The network between gut microbiota, metabolites and function and MetS clinical indicators. Visualization of the correlation network according to Spearman correlation analysis data and illustrated by Cytoscape. Node: triangle represents MetS clinical indicators, diamond represents microbes, circle dot represents metabolites, and hexagon represented functions. Red represents upregulated and blue represents down regulated, the disk sizes show the ratio of abundance in MetS over None. Edge: Red (blue) connection represents positive (negative) correlation (Spearman correlation test, FDR < 0.05), the line widths show the absolute values of coefficients. Detailed information is shown in Table S3

Spearman’s correlation analysis between fecal metabolites and clinical parameters revealed that MetS severity-dependent metabolites were significantly correlated with serum levels of diastolic pressure, TG, BMI, waist, and HDL-C (Fig. 4a). Additionally, the use of drugs for hypertension and diabetes seriously affects the BP and fast blood glucose value, so the diagnostic indicators, including fast blood glucose, systolic pressure, diastolic pressure, diabetes, and hypertension performed a strong trend consistency with the rest indicators although only a few metabolites have a statistically significant correlation. The result suggested that the metabolites may be involved in but not limited to blood pressure regulation and lipid metabolism regulation in MetS aggravation.

### Gut microbiota, metabolites and MetS-related signatures network analysis

The cross-omics correlation-based network were analyzed based on spearman correlation coefficients. It revealed that metabolites were strongly tied with both gut microbiota genera and host MetS-related signatures, especially those indicators related to lipid metabolism disorders such as TG, HDL-C and BMI (Fig. 4b and Table S3). Metabolites involved in secondary bile acids and most of glycerophospholipids, with high ratio in MetS, have tightly relationship with TG and BMI. Furthermore, biological correlations between the changed bacteria and metabolites were built based on the Sankey network analysis on Metorigin (http://metorigin.met-bioinformatics.cn/) (Figure S3), which could be the supplementary correlations between gut microbiota genera and metabolites. Notably, the metabolites were mainly involved in lipid metabolism disorder such as glycerophospholipid metabolism, glycerolipid metabolism, sphingolipid metabolism and fatty acid degradation, and so did the pathways enrich in Figure S3. Metabolites connected density between genera and clinical indicators, suggesting that metabolites were the bridge of the host–microorganism network in MetS, and lipid metabolism disorder may be a priority to uncover the mechanism of MetS.

### MetS diagnosed and prediction based on microbiota genera and metabolites

**Fig. 5:**
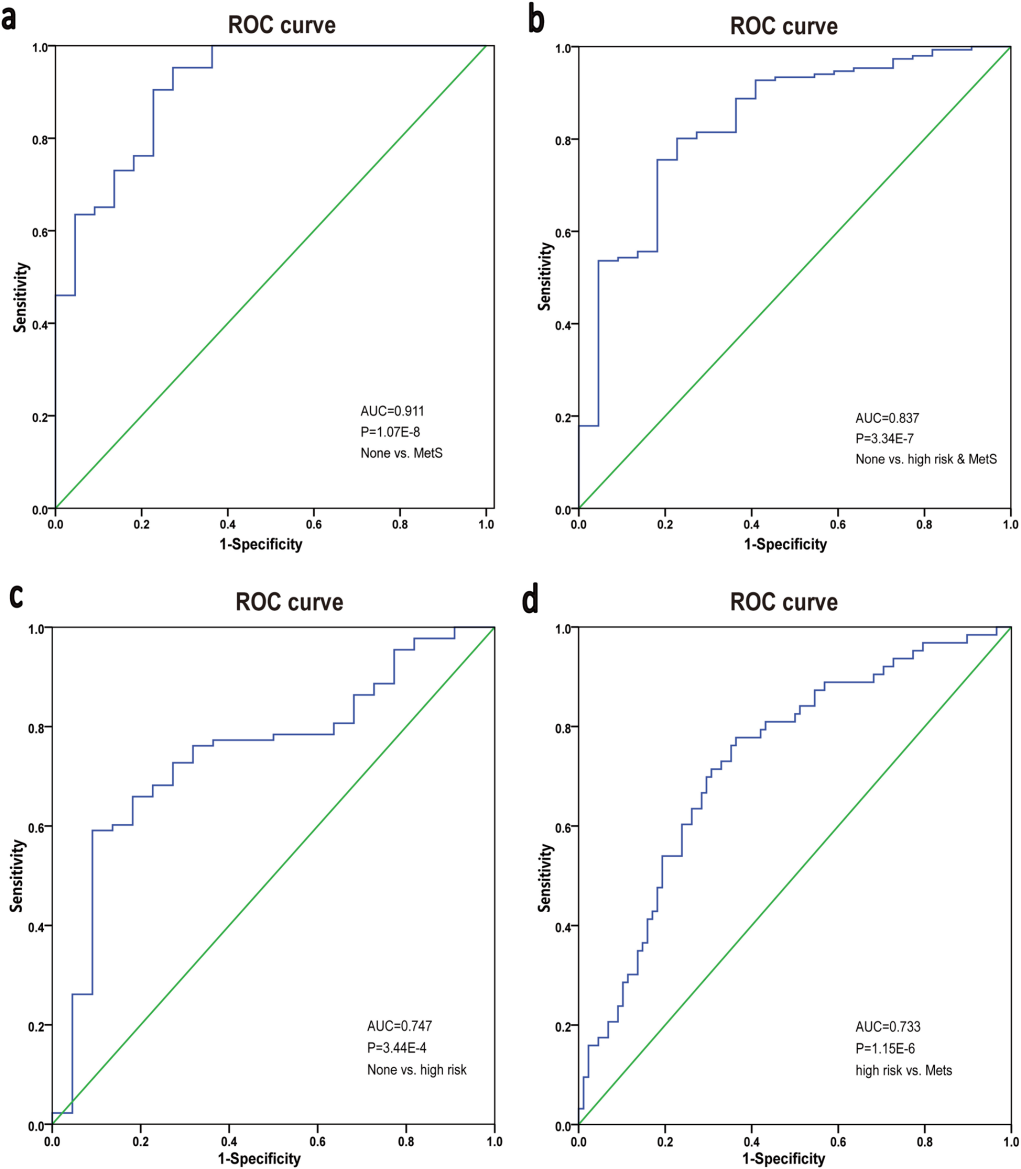
Diagnostic predictive indicators via ROC curves by logistic regression analysis. **a** Employing the combination of 5 metabolites-genera panel (*Senegalimassilia*, sphinganine, dihomo-gamma- linolenoylcholine, isodeoxycholic acid, MG (0:0/22:5/0:0)) for discriminating the MetS individuals from the None group. **b** 5 combined markers (*Senegalimassilia*, 3-Hydroxytetradecanoyl carnitine, DG[TXB2/0:0/2:0], isodeoxycholic acid, and Methylsuccinic acid) in None vs. High risk and MetS. **c** 2 combined markers (3-Hydroxytetradecanoyl carnitine and DG(TXB2/0:0/2:0)) for early diagnosis of high-risk of MetS form None. **d** 3 combined markers (L-Proline, L-palmitoylcarnitine and sphinganine) in High risk vs. MetS discrimination

To determine whether the gut genera-metabolites modules could distinguish MetS from None and even from very early stage, we established logistic regression model based on 21 metabolites and 9 genera. It was easy to distinguish MetS from the None group accurately by a microbial-metabolites panel (*Senegalimassilia*, sphinganine, dihomo-gamma-linolenoylcholine, isodeoxycholic acid, and MG [0:0/22:5/0:0]). The fecal genera-metabolites combined markers provided a large improvement in discriminating MetS with an AUC value up to 0.911 in the ROC curve, and also showed a significantly improved discrimination efficiency between None vs. High risk and MetS (AUC = 0.837, 1 genus and 4 metabolites) (Fig. 5a, b) compared to the use of microbiome biomarkers alone in Fig. 2. Therefore, the microbiota-metabolites combination analysis could optimize the accuracy of determining MetS then one-sided markers, and the combined biomarkers may provide novel insights for MetS prevention and intervention.

Further subgroup discrimination revealed that the features with predictive value include two metabolites (3-Hydroxytetradecanoyl carnitine and DG[TXB2/0:0/2:0]) in None vs. High risk and L-Proline, L-palmitoylcarnitine and sphinganine in High risk vs. MetS (Fig. 5** c**,** d**). Microbial genus marker appeared when MetS been definite diagnosis, while metabolites panel appeared in the discrimination between subgroups of disease early development, suggesting that metabolites, especially acylcarnitines and glycerolipids (mainly MG and DG), may be sensitive in reflecting the aggravation of MetS in early stage, while *Senegalimassilia* and isodeoxycholic acid appeared when those high-risk group were about developed into MetS. So, we inferred that microbial prefer to act on MetS through the affection of co-metabolites and microbial-metabolite exhibit a great ability to identify MetS and could diagnosis in an early stage.

## Discussion

The current study demonstrated that the gut microbiota and metabolites exhibited significant changes during MetS development. Multi omics analysis revealed that genera and metabolites which mainly related to lipid metabolism were disturbed accompanied by the emergence of MetS phenotype. Besides, the microbial-metabolites panel (*Senegalimassilia*, sphinganine, dihomo-gamma-linolenoylcholine, isodeoxycholic acid, and MG [0:0/22:5/0:0]) facilitate the identification of MetS in SCZ than microbial alone. Third, *Senegalimassilia* together with second bile acids, glycerolipids and acylcarnitines were more sensitive to reflect the aggravation of MetS, and *Senegalimassilia* and isodeoxycholic acid, glycerolipids and acylcarnitines might be used as biomarkers for early MetS diagnosis and reflected the severity of MetS.

Firstly, *Senegalimassilia*, which acts as the only genus for effectively distinguishing MetS, is downregulated with MetS aggravation and has manifold effects. Some reports showed that *Senegalimassilia* was down-regulated in children with overweight, it was inversely associated with inflammation markers and hepato-visceral fat [31, 32]. In addition, rats had higher abundance of gut *Senegalimassilia* when alleviated type 2 diabetes [33], indicating that down-regulated *Senegalimassilia* may have important roles on overweight and type 2 diabetes that contributes to MetS.

Among the panel, glycerolipids like diacylglycerol (DG) and monoacylglycerol (MG) were the important indicators of early MetS diagnosis. Generally, TG in the small intestine was synthesized from DG and MG by diacylglycerol acyltransferase (DGAT) and monoacylglycerol acyltransferase (MGAT) [34]. Although DG in mouse liver increased under a high-fat diet and was accompanied by hepatic insulin resistance and glucose tolerance [35, 36], it may differentiated from the trend in fecal. In our study, serum TG increased while MG and DG, acted as the precursors, decreased in fecal with the aggravation of MetS. This may infer that TG biosynthesis was promoted, and DGAT and MGAT should be further explored to confirm this process. Interestingly, sphinganine and acylcarnitine were also downregulated in fecal with MetS aggravation. Sphinganine belongs to sphingolipids together with its downstream product ceramides (Cer). As a class of cometabolites between host and microbiome, sphingolipids have well-defined roles in mammalian energy metabolism pathways, acting as cell adhesion molecules to regulate cell proliferation, differentiation, and apoptosis [37, 38]. Reportedly, Cers elevated in liver have been reported to correlate with hepatic insulin resistance and associated with increased total DG and TG [39]. However, they also exhibit the reverse trend in gut contents because of a complicated process from gut to blood and organs.

This is similar with acylcarnitine, as their function and role in MetS pathophysiology are not completely clarified. We found that 3-Hydroxytetradecanoyl carnitine (C14-OH) decreased with MetS severity. It is said that long-chain (C14-C18) acylcarnitines decreased in the plasma of patients with SCZ than healthy people, which may be influenced by different transporters in the plasma membrane [40], and was found to increase antioxidant activity, and regulate immune functions [41]. However, more certain findings have emphasized that acylcarnitine content was influenced by microbiome-derived metabolite δ-valerobetaine, which decreased long-chain acylcarnitine consumption in mitochondrial β-oxidation to accumulate tissue fat [42]. Acylcarnitines are harmful fatty acid intermediates, especially those with saturated fatty acids, which have a role in inducing oxidative phosphorylation inhibition and increasing apoptosis [43]. This was consistent with our result that palmitoylcarnitine (C16:0) and stearoylcarnitine (C18:0) increased in patients with SCZ with MetS aggravation, acting as the markers to reflect different metabolic conditions. Furthermore, population-based studies revealed increased palmitoylcarnitine (C16:0) and stearoylcarnitine (C18:0) in SCZ with MetS compared with NoMetS [14, 44], demonstrated that long-chain carnitines with saturated fatty acids were potentially associated with insulin resistance, incomplete fatty acid oxidation, and mitochondrial lipid overload and played positive roles in the development of MetS.

## Limitations

Some possible confounds are worth mentioning. Firstly, many patients were prescribed not only antipsychotic medication but also other medications, including drugs for hypertension, diabetes, and others. Fortunately, PLS-DA plots demonstrate no separation between PMs-only and PMs with other kinds of drugs in three different groups both in positive and negative ion mode (Table 1,Figure S4). This indicates that the differences in medication quantity do not significantly influence the grouping, and other supplementary treatments were not prominent confounding variables affecting our result interpretation. Additionally, the sample size in our study was somewhat small, and another fully matched cohort is required for external validation of the findings. Finally, further experimental explanation of the causal relationship between the changes in bacteria and metabolites and MetS would be beneficial. Nonetheless, our study provides a novel framework for mapping the role of gut microbiota and metabolites in anti-psychiatric medicine-induced MetS.

## Conclusion

In summary, it is urgent that significant and convenient biomarkers should be identified to monitor vulnerable subjects from no risk indicators to MetS due to the high incidence and high risk of MetS in SCZ. Following the use of combined omics for early diagnosis, we discovered that specific microbiota and metabolites are interrelated and influenced each other, and the microbial-metabolites panel demonstrated a strong discriminatory ability in diagnosing MetS. What is more, the disturbed biomarkers, primarily associated with lipid metabolism, were more sensitive in reflecting the exacerbation of MetS, helping to uncover the mechanism of MetS induced by antipsychotics. Overall, these findings contribute new insights into the role of gut microbiota and metabolites in early clinical diagnosis of MetS in SCZ. Furthermore, it revealed novel potential etiologies for MetS in patients with SCZ, and the changed molecular and functions may be the potential targets to treat or ameliorate MetS in SCZ.

### Supplementary Information

Supplementary Material 1.

## Data Availability

Data is provided within the manuscript or supplementary information files, and any research data required for reanalysis is available upon request.

## Abbreviations

SCZ: Schizophrenia
MetS: Metabolic syndrome
AUC: Area under the curve
SGAs: Second-generation antipsychotics
BP: Blood pressure
HDL-C: High-density lipoprotein cholesterol
TG: Triglycerides
CVD: Cardiovascular diseases
SCFAs: Short-chain fatty acids
LPS: Lipopolysaccharide
ICD-10: The tenth edition of the International Classification of Diseases
OTUs: Operational taxonomic units
KEGG: Kyoto Encyclopedia of Genes and Genomes
IPA: Ingenuity Pathway Analysis
ROC: Receiver operating characteristic
PANSS: Positive and Negative Syndrome Scale
PMs: Psychiatry medication quantity
PCA: Principal component analysis

## References

[1] Haddad PM, Correll CU. The acute efficacy of antipsychotics in schizophrenia: a review of recent meta-analyses. Ther Adv Psychopharmacol. 2018;8(11):303–18. 10.1177/2045125318781475. 30344997 10.1177/2045125318781475 PMC6180374

[2] Siskind D, McCartney L, Goldschlager R, Kisely S. Clozapine v. first- and second-generation antipsychotics in treatment-refractory schizophrenia: systematic review and meta-analysis. Br J Psychiatry. 2016;209(5):385–92. 10.1192/bjp.bp.115.177261. 27388573 10.1192/bjp.bp.115.177261

[3] Marder SR, Cannon TD. Schizophrenia. N Engl J Med. 2019;381(18):1753–61. 10.1056/NEJMra1808803. 31665579 10.1056/NEJMra1808803

[4] Pillinger T, McCutcheon RA, Vano L, Mizuno Y, Arumuham A, Hindley G, et al. Comparative effects of 18 antipsychotics on metabolic function in patients with schizophrenia, predictors of metabolic dysregulation, and association with psychopathology: a systematic review and network meta-analysis. Lancet Psychiatry. 2020;7(1):64–77. 10.1016/s2215-0366(19)30416-x. 31860457 10.1016/s2215-0366(19)30416-x PMC7029416

[5] Penninx B, Lange SMM. Metabolic syndrome in psychiatric patients: overview, mechanisms, and implications. Dialogues Clin Neurosci. 2018;20(1):63–73. 10.31887/DCNS.2018.20.1/bpenninx. 29946213 10.31887/DCNS.2018.20.1/bpenninx PMC6016046

[6] [Clinical guidelines for prevention and treatment of type 2 diabetes mellitus in the elderly in China (2022 edition)]. Zhonghua nei ke za zhi. 2022;61(1):12–50. 10.3760/cma.j.cn112138-20211027-00751. 10.3760/cma.j.cn112138-20211027-00751 34979769

[7] Howes OD, Bhatnagar A, Gaughran FP, Amiel SA, Murray RM, Pilowsky LS. A prospective study of impairment in glucose control caused by clozapine without changes in insulin resistance. Am J Psychiatry. 2004;161(2):361–3. 10.1176/appi.ajp.161.2.361. 14754788 10.1176/appi.ajp.161.2.361 PMC3685269

[8] Huhn M, Nikolakopoulou A, Schneider-Thoma J, Krause M, Samara M, Peter N, et al. Comparative efficacy and tolerability of 32 oral antipsychotics for the acute treatment of adults with multi-episode schizophrenia: a systematic review and network meta-analysis. Lancet (London, England). 2019;394(10202):939–51. 10.1016/s0140-6736(19)31135-3. 31303314 10.1016/s0140-6736(19)31135-3 PMC6891890

[9] Grundy SM, Cleeman JI, Daniels SR, Donato KA, Eckel RH, Franklin BA, et al. Diagnosis and management of the metabolic syndrome: an American Heart Association/National Heart, Lung, and Blood Institute Scientific Statement. Circulation. 2005;112(17):2735–52. 10.1161/circulationaha.105.169404. 16157765 10.1161/circulationaha.105.169404

[10] Correll CU, Solmi M, Veronese N, Bortolato B, Rosson S, Santonastaso P, et al. Prevalence, incidence and mortality from cardiovascular disease in patients with pooled and specific severe mental illness: a large-scale meta-analysis of 3,211,768 patients and 113,383,368 controls. World Psychiatry. 2017;16(2):163–80. 10.1002/wps.20420. 28498599 10.1002/wps.20420 PMC5428179

[11] Newcomer JW. Antipsychotic medications: metabolic and cardiovascular risk. J Clin Psychiatry. 2007;68(Suppl 4):8–13. 17539694

[12] Bora E, Akdede BB, Alptekin K. The relationship between cognitive impairment in schizophrenia and metabolic syndrome: a systematic review and meta-analysis. Psychol Med. 2017;47(6):1030–40. 10.1017/s0033291716003366. 28032535 10.1017/s0033291716003366

[13] Lindenmayer JP, Khan A, Kaushik S, Thanju A, Praveen R, Hoffman L, et al. Relationship between metabolic syndrome and cognition in patients with schizophrenia. Schizophr Res. 2012;142(1–3):171–6. 10.1016/j.schres.2012.09.019. 23106932 10.1016/j.schres.2012.09.019

[14] Cao B, Chen Y, McIntyre RS, Yan LL. Acyl-Carnitine plasma levels and their association with metabolic syndrome in individuals with schizophrenia. Psychiatry Res. 2020;293: 113458. 10.1016/j.psychres.2020.113458. 32977055 10.1016/j.psychres.2020.113458

[15] Burghardt KJ, Ellingrod VL. Detection of Metabolic Syndrome in Schizophrenia and Implications for Antipsychotic Therapy Is There a Role for Folate? Mol Diagn Ther. 2013;17(1):21–30. 10.1007/s40291-013-0017-8. 23341251 10.1007/s40291-013-0017-8 PMC4077272

[16] Sanna S, van Zuydam NR, Mahajan A, Kurilshikov A, Vila AV, Vosa U, et al. Causal relationships among the gut microbiome, short-chain fatty acids and metabolic diseases. Nat Genet. 2019;51(4):600-+. 10.1038/s41588-019-0350-x. 30778224 10.1038/s41588-019-0350-x PMC6441384

[17] Khasanova AK, Dobrodeeva VS, Shnayder NA, Petrova MM, Pronina EA, Bochanova EN, et al. Blood and Urinary Biomarkers of Antipsychotic-Induced Metabolic Syndrome. Metabolites. 2022;12(8). 10.3390/metabo12080726. 10.3390/metabo12080726 PMC9416438 36005598

[18] Depommier C, Everard A, Druart C, Plovier H, Van Hul M, Vieira-Silva S. Supplementation with Akkermansia muciniphila in overweight and obese human volunteers: a proof-of-concept exploratory study. Nat Med. 2019;25(7):1096–103. 10.1038/s41591-019-0495-2. 31263284 10.1038/s41591-019-0495-2 PMC6699990

[19] Vijay-Kumar M, Aitken JD, Carvalho FA, Cullender TC, Mwangi S, Srinivasan S, et al. Metabolic syndrome and altered gut microbiota in mice lacking Toll-like receptor 5. Science (New York, NY). 2010;328(5975):228–31. 10.1126/science.1179721. 10.1126/science.1179721 PMC4714868 20203013

[20] Dabke K, Hendrick G, Devkota S. The gut microbiome and metabolic syndrome. J Clin Investig. 2019;129(10):4050–7. 10.1172/jci129194. 31573550 10.1172/jci129194 PMC6763239

[21] Matey-Hernandez ML, Williams FMK, Potter T, Valdes AM, Spector TD, Menni C. Genetic and microbiome influence on lipid metabolism and dyslipidemia. Physiol Genomics. 2018;50(2):117–26. 10.1152/physiolgenomics.00053.2017. 29341867 10.1152/physiolgenomics.00053.2017 PMC5867613

[22] Liu H, Chen X, Hu X, Niu H, Tian R, Wang H, et al. Alterations in the gut microbiome and metabolism with coronary artery disease severity. Microbiome. 2019;7(1):68. 10.1186/s40168-019-0683-9. 31027508 10.1186/s40168-019-0683-9 PMC6486680

[23] Franzosa EA, Sirota-Madi A, Avila-Pacheco J, Fornelos N, Haiser HJ, Reinker S, et al. Gut microbiome structure and metabolic activity in inflammatory bowel disease. Nat Microbiol. 2019;4(2):293–305. 10.1038/s41564-018-0306-4. 30531976 10.1038/s41564-018-0306-4 PMC6342642

[24] Chen S, Zhou Y, Chen Y, Gu J. fastp: an ultra-fast all-in-one FASTQ preprocessor. Bioinformatics (Oxford, England). 2018;34(17):i884–90. 10.1093/bioinformatics/bty560. 30423086 10.1093/bioinformatics/bty560 PMC6129281

[25] Magoč T, Salzberg SL. FLASH: fast length adjustment of short reads to improve genome assemblies. Bioinformatics (Oxford, England). 2011;27(21):2957–63. 10.1093/bioinformatics/btr507. 21903629 10.1093/bioinformatics/btr507 PMC3198573

[26] Edgar RC. UPARSE: highly accurate OTU sequences from microbial amplicon reads. Nat Methods. 2013;10(10):996–8. 10.1038/nmeth.2604. 23955772 10.1038/nmeth.2604

[27] Wang Q, Garrity GM, Tiedje JM, Cole JR. Naive Bayesian classifier for rapid assignment of rRNA sequences into the new bacterial taxonomy. Appl Environ Microbiol. 2007;73(16):5261–7. 10.1128/aem.00062-07. 17586664 10.1128/aem.00062-07 PMC1950982

[28] Zhao H, Du H, Liu M, Gao S, Li N, Chao Y, et al. Integrative proteomics-metabolomics strategy for pathological mechanism of vascular depression mouse model. J Proteome Res. 2018;17(1):656–69. 10.1021/acs.jproteome.7b00724. 29190102 10.1021/acs.jproteome.7b00724

[29] Yu G, Xu C, Zhang D, Ju F, Ni Y. MetOrigin: Discriminating the origins of microbial metabolites for integrative analysis of the gut microbiome and metabolome. iMeta. 2022;1(1):e10. 10.1002/imt2.10. 38867728 10.1002/imt2.10 PMC10989983

[30] Lim K, Peh OH, Yang ZX, Rekhi G, Rapisarda A, See YM, et al. Large-scale evaluation of the Positive and Negative Syndrome Scale (PANSS) symptom architecture in schizophrenia. Asian J Psychiatr. 2021;62:102732. 10.1016/j.ajp.2021.102732. 34118560 10.1016/j.ajp.2021.102732

[31] Adamberg K, Adamberg S, Emits K, Larionova A, Voor T, Jaagura M, et al. Composition and metabolism of fecal microbiota from normal and overweight children are differentially affected by melibiose, raffinose and raffinose-derived fructans. Anaerobe. 2018;52:100–10. 10.1016/j.anaerobe.2018.06.009. 29935270 10.1016/j.anaerobe.2018.06.009

[32] Garcia-Beltran C, Malpique R, Carbonetto B, Gonzalez-Torres P, Henares D, Brotons P, et al. Gut microbiota in adolescent girls with polycystic ovary syndrome: effects of randomized treatments. Pediatr Obes. 2021;16(4):e12734. 10.1111/ijpo.12734. 32989872 10.1111/ijpo.12734

[33] Gao K, Yang R, Zhang P, Wang ZY, Jia CX, Zhang FL, et al. Effects of Qijian mixture on type 2 diabetes assessed by metabonomics, gut microbiota and network pharmacology. Pharmacol Res. 2018;130:93–109. 10.1016/j.phrs.2018.01.011. 29391233 10.1016/j.phrs.2018.01.011

[34] Yang M, Nickels JT. MOGAT2: A New Therapeutic Target for Metabolic Syndrome. Diseases. 2015;3(3):176–92. 10.3390/diseases3030176. 28943619 10.3390/diseases3030176 PMC5548241

[35] Choi CS, Savage DB, Kulkarni A, Yu XX, Liu ZX, Morino K, et al. Suppression of diacylglycerol acyltransferase-2 (DGAT2), but not DGAT1, with antisense oligonucleotides reverses diet-induced hepatic steatosis and insulin resistance. J Biol Chem. 2007;282(31):22678–88. 10.1074/jbc.M704213200. 17526931 10.1074/jbc.M704213200

[36] Turner N, Kowalski GM, Leslie SJ, Risis S, Yang C, Lee-Young RS, et al. Distinct patterns of tissue-specific lipid accumulation during the induction of insulin resistance in mice by high-fat feeding. Diabetologia. 2013;56(7):1638–48. 10.1007/s00125-013-2913-1. 23620060 10.1007/s00125-013-2913-1

[37] Summers SA, Chaurasia B. Metabolic Messengers: ceramides. Nat Metab. 2019;1(11):1051–8. 10.1038/s42255-019-0134-8. 32694860 10.1038/s42255-019-0134-8 PMC7549391

[38] Wasserman AH, Venkatesan M. Bioactive Lipid Signaling in Cardiovascular Disease, Development, and Regeneration. Cells. 2020;9(6):1391. 10.3390/cells9061391. 32503253 10.3390/cells9061391 PMC7349721

[39] Jornayvaz FR, Birkenfeld AL, Jurczak MJ, Kanda S, Guigni BA, Jiang DC, et al. Hepatic insulin resistance in mice with hepatic overexpression of diacylglycerol acyltransferase 2. Proc Natl Acad Sci U S A. 2011;108(14):5748–52. 10.1073/pnas.1103451108. 21436037 10.1073/pnas.1103451108 PMC3078388

[40] Cao B, Wang D, Pan Z, Brietzke E, McIntyre RS, Musial N, et al. Characterizing acyl-carnitine biosignatures for schizophrenia: a longitudinal pre- and post-treatment study. Transl Psychiatry. 2019;9(1):19. 10.1038/s41398-018-0353-x. 30655505 10.1038/s41398-018-0353-x PMC6336814

[41] Waagsbo B, Svardal A, Ueland T, Landro L, Oktedalen O, Berge RK, et al. Low levels of short- and medium-chain acylcarnitines in HIV-infected patients. Eur J Clin Invest. 2016;46(5):408–17. 10.1111/eci.12609. 26913383 10.1111/eci.12609

[42] Liu KH, Owens JA. Microbial metabolite delta-valerobetaine is a diet-dependent obesogen. Nat Metab. 2021;3(12):1694–705. 10.1038/s42255-021-00502-8. 34931082 10.1038/s42255-021-00502-8 PMC8711632

[43] Liepinsh E, Makrecka-Kuka M, Volska K, Kuka J, Makarova E, Antone U, et al. Long-chain acylcarnitines determine ischaemia/reperfusion-induced damage in heart mitochondria. Biochem J. 2016;473(9):1191–202. 10.1042/BCJ20160164. 26936967 10.1042/BCJ20160164

[44] Sun L, Liang L, Gao X, Zhang H, Yao P, Hu Y, et al. Early Prediction of Developing Type 2 Diabetes by Plasma Acylcarnitines: A Population-Based Study. Diabetes Care. 2016;39(9):1563–70. 10.2337/dc16-0232. 27388475 10.2337/dc16-0232

