# Supplementary material for: Gut microbiome and metabolism alterations in schizophrenia with metabolic syndrome severity
Source: BMC Psychiatry. 2024 Jul 24;24:529. doi: 10.1186/s12888-024-05969-9 (PMC11267952; doi:10.1186/s12888-024-05969-9)
Supplement: Supplementary file 1 — Supplementary Material 1. [file 12888_2024_5969_MOESM1_ESM.pdf]

Supplementary Information

Title: Gut microbiome and metabolism alterations in schizophrenia with metabolic syndrome severity

Running head: Early diagnosis of MetS in SCZ based on multi-omics study

Hongxia Zhao[1,2*], Guang Zhu[3*], Tong Zhu [1,4*], Binbin Ding[3], Ahong Xu[3], Songyan Gao[5], Yufan Chao[1], Na Li[1], Yongchun Chen[6], Zuowei Wang[3,7‡], Yong Jie[3,7‡], Xin Dong[1,7‡]

[1] School of medicine, Shanghai University, Shanghai, 200444, China.

[2] Zhanjiang institute of clinical medicine, Central People's Hospital of Zhanjiang, Zhanjiang, 524045, China.

[3] Hongkou Mental Health Center, Shanghai, 200083, China.

[4] School of Life Sciences, Shanghai University, Shanghai 200444, China.

[5] Institute of translational medicine, Shanghai University, Shanghai， 200444, China.

[6] Department of pharmacy, The First Naval Hospital of Southern Theater Command, Zhanjiang, 524000, China.

[7] Clinical Research Center for Mental Health, School of Medicine, Shanghai University, Shanghai 20083, China

[*] These authors contributed equally to this work

[‡]Corresponding author, E-mail: wzwhk@163.com (Z.W.), jy96jw@163.com (Y.J.), dongxinsmmu@126.com (X.D.)

Supplementary Information

A: 16S rRNA gene amplification and purification:

The PCR amplification of 16S rRNA gene was performed as follows: initial denaturation at 95 °C for 3 min, followed by 27 cycles of denaturing at 95 °C for 30 s, annealing at 55 °C for 30 s and extension at 72 °Cfor 45 s, and single extension at 72 °C for 10 min, and end at 4 °C. The PCR mixtures contain 5 × *TransStart* FastPfu buffer 4 μL, 2.5 mM dNTPs 2 μL, forward primer (5 μM) 0.8 μL, reverse primer (5 μM) 0.8 μL, *TransStart* FastPfu DNA Polymerase 0.4 μL, template DNA 10 ng, and finally ddH_2O up to 20 μL. PCR reactions were performed in triplicate. The PCR product was extracted from 2% agarose gel and then purified using the AxyPrep DNA Gel Extraction Kit (Axygen Biosciences, Union City, CA, USA) according to manufacturer's instructions and quantified using Quantus™ Fluorometer (Promega, USA).

16S rRNA gene sequence analysis criteria:

(i) the 300 bp reads were truncated at any site receiving an average quality score of <20 over a 50 bp sliding window, and the truncated reads shorter than 50 bp were discarded, reads containing ambiguous characters were also discarded; (ii) only overlapping sequences longer than 10 bp were assembled according to their overlapped sequence. The maximum mismatch ratio of overlap region is 0.2. Reads that could not be assembled were discarded; (iii) Samples were distinguished according to the barcode and primers, and the sequence direction was adjusted, exact barcode matching, two nucleotide mismatches in primer matching.

B: The profile of metabolomics analysis:

Each sample was weighed and then homogenized in 200 μL of a pre-cooled 80% methanol solution that contained 4 μg/mL 2-chloro-l-phenylalanine as an internal standard. After vortexing for 1 min, the mixtures were centrifuged at 13,000 rpm for 15 min at 4 °C, and 150 μL of supernatant were added to the sample vials. A quality control sample (QC) was prepared by mixing aliquots from all supernatant samples (10 μL from each sample).

Chromatographic column was Waters ACQUITY UPLC HSS T3 analytical column (2.1 mm x100 mm, 1.8 μm, Waters, Milford, Massachusetts). The column temperature was 40 °C and the sample chamber temperature was set at 4 °C. The mobile phase in this experiment was acetonitrile (with 0.1% formic acid, eluent A) and water with 5% ACN and 0.1% formic acid (eluent B) at 0.4

mL/min. The injection volume was 4 μL. All the samples were injected randomly and QC samples were analyzed once every eighteen injections to assure the system stability. The mobile phase gradient started with 100% B for 2 minutes and drop to 50% B within 3 minutes, then down to 15% B at the 13th minute, after that, B continue to fall to 5% within 1 minute and keep holding for 1 minute. Mass spectrometric conditions were set in both positive and negative ion modes with key parameter settings as follow: mass range was between 100 to 1100 m/z, fragmentor set at 120 V, gas temperature was at 350ºC, the flow rate of drying gas was 11 L/min, nebulizer set at 45 psig, V, capillary was 3.5 kV in positive ion mode and 3.2 kV in negative ion mode.

The raw UHPLC–MS data were transformed into a common data file format (. mzdata) using MassHunter Qualitative software. The interferences of the isotopes were excluded and the absolute peak height was set at 300 counts. The XCMS program was applied for peak extraction, peak alignment, and automatic integration. The 80% rule was used to filter the ions and the remaining ions were normalized by both internal standard peak area and the weight of each sample.

Various metabolites that are responsible for the discrimination between groups were filtered by SIMCA-P software (version 14.1, Umetrics AB, Umeå, Sweden) and SPSS. Specific metabolites selected as biomarker candidates for further statistical analysis when identified with the significance threshold of variable importance (VIP, a value which shows the contribution of the variable to the difference between the groups) of the PLS-DA model of >1.0 and the nonparametric univariate method (Mann–Whitney U test) with P-values of <0.05.

To investigate the related disturbed pathways and function, we performed the differential metabolites using MetaboAnalyst 5.0 platform and QIAGEN's Ingenuity Pathway Analysis (IPA®, QIAGEN Redwood City, www.qiagen.com/ingenuity). The P value and z-score were used to evaluate IPA results. The P value was obtained from the right-tailed Fisher's exact test algorithm, which shows whether the association between a set of meaningful molecules in the experiment and the known process/pathway/transcription comes from random matching. Neither the effect of molecules nor fold change between molecules in the data set was taken into account in the process of analysis. The z-score evaluated the effect of molecular changes on biological processes. In general, a z-score > 2 suggests that the corresponding molecules/functions are significantly activated, and a z-score ≤ 2 indicates significant inhibition.

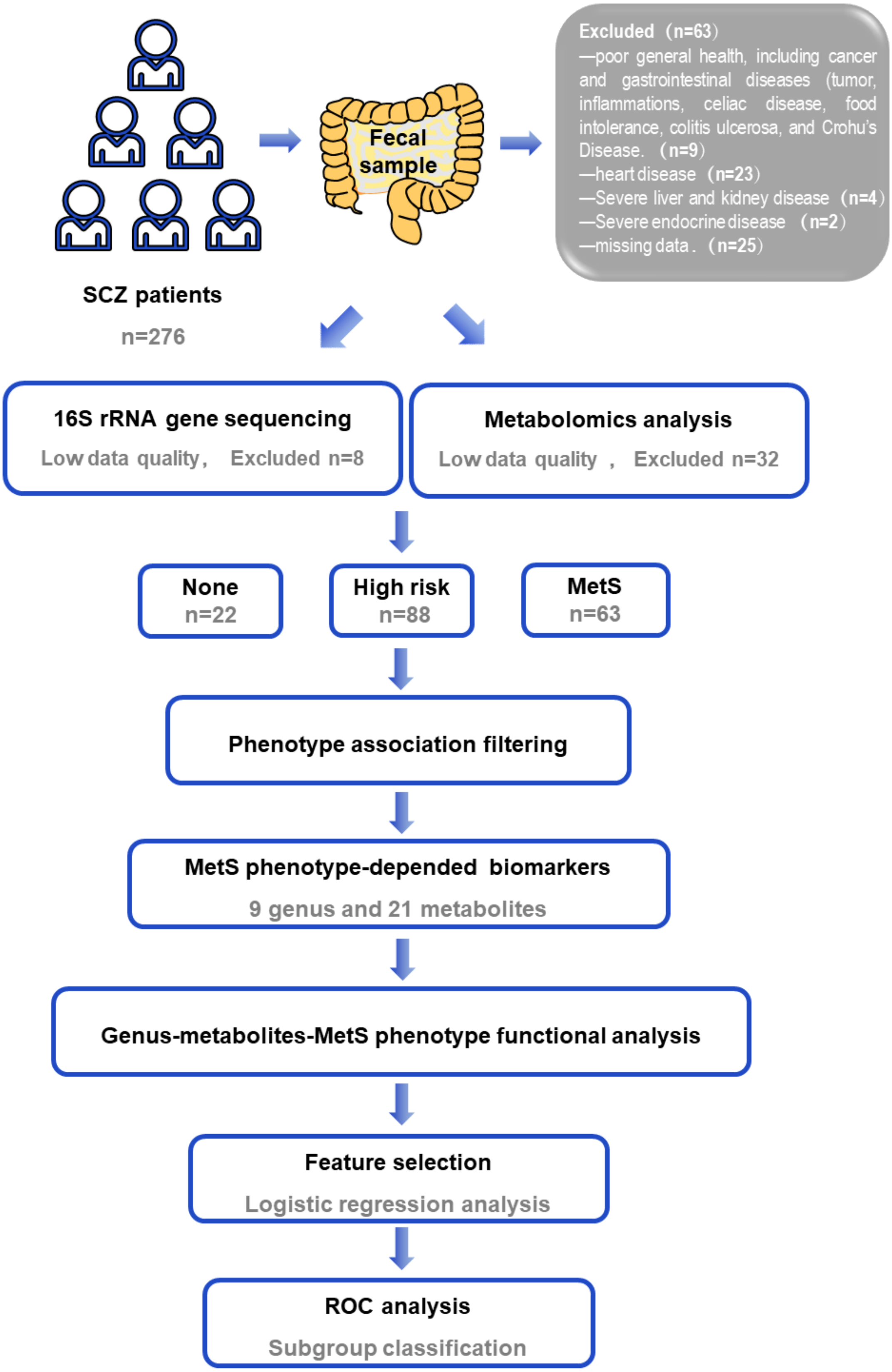

Figure S1. The workflow diagram of this work.

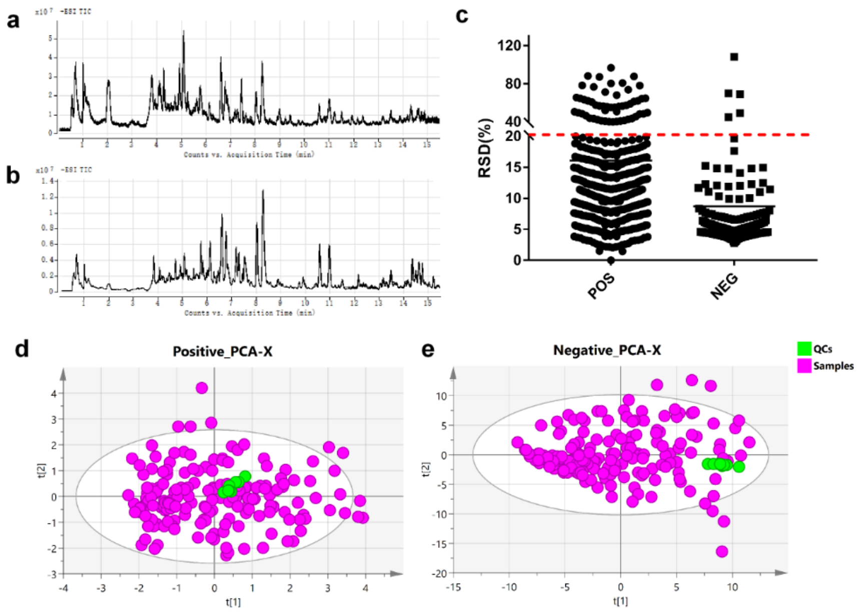

Figure S2. The evaluation of QC stability in positive and negative ion mode. (**a, b**) The overlapped total ion chromatography (TIC) of 10 QC samples, **(c)** RSD values of the peak intensities in QC samples, more than 80% of the RSD values of the QC samples were less than 20%. **(d,e)** PCA score plot of QCs and samples in ESI positive and negative ion mode, respectively.

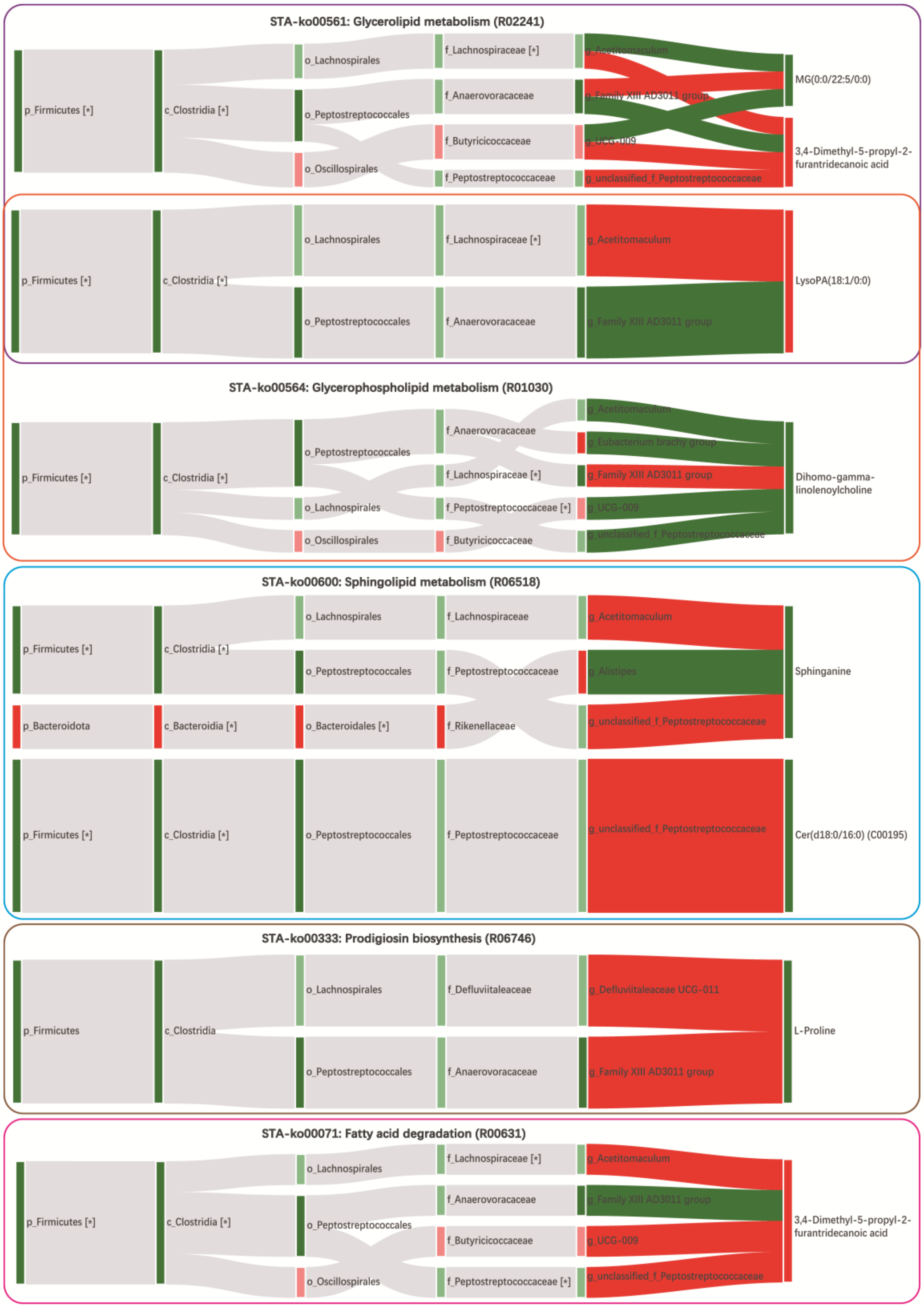

Figure S3. Gut microbiota, metabolites and their biological functions in SCZ with MetS. Red (green) bars indicate significantly up-regulated (down-regulated) microbes or metabolites, Red (green) bands indicate significantly positive (negative) correlation (red, R>0 and P<0.05, green, R<0 and P<0.05).

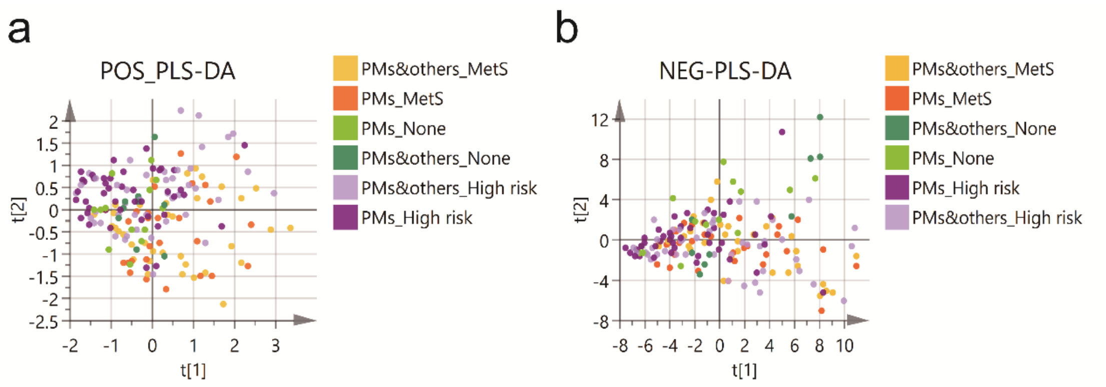

Figure S4. Impact of other drugs on global metabolism phenotypes. (**a**) PLS-DA plot in positive ion mode, (**b**) PLS-DA plot in negative ion mode.
